# Adherence to clinical guidelines is associated with reduced inpatient mortality among children with severe anemia in Ugandan hospitals

**DOI:** 10.1371/journal.pone.0210982

**Published:** 2019-01-25

**Authors:** Robert O. Opoka, Andrew S. Ssemata, William Oyang, Harriet Nambuya, Chandy C. John, Charles Karamagi, James K. Tumwine

**Affiliations:** 1 Department of Paediatrics and Child Health, College of Health Sciences, Makerere University, Kampala, Uganda; 2 Department of Psychiatry, College of Health Sciences, Makerere University, Kampala, Uganda; 3 Children’s Ward, Lira Regional Referral Hospital, Lira, Uganda; 4 Nalufenya Children’s Ward, Jinja Regional Referral Hospital, Jinja, Uganda; 5 Ryan White Center for Pediatric Infectious Disease and Global Health, Indiana University School of Medicine, Indianapolis, Indiana, United States of America; Universidade Nova de Lisboa Instituto de Higiene e Medicina Tropical, PORTUGAL

## Abstract

**Background:**

In resource limited settings, there is variability in the level of adherence to clinical guidelines in the inpatient management of children with common conditions like severe anemia. However, there is limited data on the effect of adherence to clinical guidelines on inpatient mortality in children managed for severe anemia.

**Methods:**

We analyzed data from an uncontrolled before and after in-service training intervention to improve quality of care in Lira and Jinja regional referral hospitals in Uganda. Inpatient records of children aged 0 to 5 years managed as cases of ‘severe anemia (SA)’ were reviewed to ascertain adherence to clinical guidelines and compare inpatient deaths in SA children managed versus those not managed according to clinical guidelines. Logistic regression analysis was conducted to evaluate the relationship between clinical care factors and inpatient deaths amongst patients managed for SA.

**Results:**

A total of 1,131 children were assigned a clinical diagnosis of ‘severe anemia’ in the two hospitals. There was improvement in the level of care after the in-service training intervention with more children being managed according to clinical guidelines compared to the period before, 218/510 (42.7%) vs 158/621 (25.4%) (p < 0.001). Overall, children managed according to clinical guidelines had reduced risk of inpatient mortality compared to those not managed according to clinical guidelines, [OR 0.28, (95%, CI 0.14, 0.55), p = 0.001]. Clinical care factors associated with decreased risk of inpatient death included, having pre-transfusion hemoglobin done to confirm diagnosis [OR 0.5; 95% CI 0.29, 0.87], a co-morbid diagnosis of severe malaria [OR 0.4; 95% CI 0.25, 0.76], and being reviewed after admission by a clinician [OR 0.3; 95% CI 0.18, 0.59], while a co-morbid diagnosis of severe acute malnutrition was associated with increased risk of inpatient death [OR 4.2; 95% CI 2.15, 8.22].

**Conclusion:**

Children with suspected SA who are managed according to clinical guidelines have lower in-hospital mortality than those not managed according to the guidelines. Efforts to reduce inpatient mortality in SA children in resource-limited settings should focus on training and supporting health workers to adhere to clinical guidelines.

## Introduction

Severe anemia (SA) is a common cause of childhood morbidity and mortality in resource-limited settings. It accounts for 9–29% of total pediatric admissions and 8–17% of hospital deaths in sub-Saharan Africa [1–6]. According to clinical guidelines from the World Health Organization [7], the management of severe anemia (SA) involves: confirmation of the diagnosis via measurement of hemoglobin (Hb) level; investigation of the specific cause of anemia via appropriate diagnostic tests (such as absolute reticulocyte count, blood smear); and prompt provision of a blood transfusion (if indicated) to correct the severe anemia and additional treatment for the specific cause of the SA [7]. However, in resource-limited settings, there are many challenges involved in the provision of care to critically ill children such as those with SA. In these settings, blood is often not available for transfusion and, when available, there is often considerable delay in receipt of blood [8, 9]. Other challenges include inadequate laboratory and clinical investigation to support/confirm the SA diagnosis and etiology, lack of essential supplies and medicines, and disregard of laboratory results by clinicians [10]. The above challenges are compounded by human resource problems such as staff shortages and lack of skills required for resuscitation of critically ill children, including patients with SA [11].

These challenges to inpatient care contribute to high variability in adherence to clinical guidelines. A recent study assessing level of care in Ugandan health facilities found that only 38% of children presenting with SA were managed appropriately [12], findings that are similar to those from other resource limited settings [13, 14]. Consequently, interventions to improve quality of inpatient care in critically-ill patient populations were proposed. In Kenya, the implementation of the in-service training program called “Emergency Triage Assessment and Treatment Plus Admission Care” (ETAT+) was found to improve adherence to clinical guidelines and quality of care provided to critically-ill children presenting to hospital [15–17]. ETAT+ has also been implemented in several health units with similar results in Rwanda and Uganda [18]. In Uganda, the implementation of ETAT+ was accompanied by the roll out of clinical guidelines for emergency paediatric inpatient care in 2012. However, the effect of adherence to clinical guidelines on inpatient outcomes in Uganda has not been described. Hence there was need to objectively determine the association between adherence to clinical guidelines and inpatient mortality of common condition such as SA. In addition, there was need to identify clinical care factors in the management of severe anemia that contribute to inpatient mortality in order to target interventions appropriately. We reviewed treatment records for patients managed as cases of severe anemia to ascertain the effect of adherence to clinical guidelines and clinical care factors on inpatient mortality in two referral hospitals in Uganda.

## Materials and methods

### Design

We reviewed inpatient records of children managed for SA from an uncontrolled before-and-after study of a quality improvement (QI) intervention.

### Study site

The study was performed at the children’s wards of Jinja and Lira Regional Referral Hospitals (RRH) located in the central and mid northern regions of Uganda. Both are public, free-for-service hospitals with capacity to manage children with SA. The hospitals were selected to represent regions of varying malaria transmission intensity. Malaria infection is a major driver of severe anemia. Jinja RRH hospital serves an area of seasonal malaria transmission intensity while Lira RRH serves an area of all year high malaria transmission [19]. Both hospitals have dedicated children’s wards. Jinja has a 60-bed pediatric inpatient capacity while Lira has a 28-bed pediatric inpatient capacity. The hospitals have laboratories with diagnostic capabilities that include: malaria blood smears, complete blood counts (CBC), stool and urine microscopy and human immunodeficiency virus (HIV) antibody testing. In Jinja, Hb measurement by Hemacue was also available. The laboratories are open every day for 24 hours. Typically routine tests are done between 8.00 am and 5.00 pm on working days while only emergency tests like grouping and cross matching for blood transfusions are done after-hours and on weekends. Laboratory results from routine tests are usually collected by ward clinicians once they are ready.

In Uganda, blood used in public and private funded health facilities is provided by the Uganda Blood Transfusion Service. Blood is obtained from voluntary, anonymous donors and sent to one of the seven regional blood transfusion centres for testing and preparation before distribution to health units for use. Lira hospital is serviced by the Lira Regional Centre and Jinja hospital is serviced by the Jinja Regional Centre. Both regional centres are located in the respective hospitals. So when blood is requested for, and is available, patients are able to receive it immediately.

### Patient flow

Children are managed by a clinical team lead by Pediatricians (1 in Lira and 3 in Jinja) and includes medical officers, intern physicians and nurses. Sick children coming to the hospital are evaluated in an outpatient setting by clinical officers (diploma level training in medicine) and sent to the pediatric wards for admission. In the pediatric wards, the patients are assessed by intern physicians who provide a 24-hour coverage of the wards and the initial management plan. The pediatrician or medical officers conduct daily ward rounds to review admitted children. All management decisions and procedures are documented in patient files. The files are kept by the ward nurse and taken to the records department at the end of hospitalization for storage and future retrieval. All inpatient deaths are recorded in death summary book that is kept on the wards. The hospitals use International Statistical Classification of Diseases and Related Health Problems 10^th^ Revision to classify diseases.

#### Clinical guidelines for management of patients with severe anemia in Uganda

In Uganda the paediatric clinical practice guideline (CPG) “Basic Paediatric protocols for Uganda” is widely accepted in hospitals and taught in medical schools as standard of pediatric inpatient care in Uganda. The guidelines were adapted from World Health Organization pocket book of hospital care for children [7] after a consensus meeting between academics, researchers and Uganda Ministry of Health officials in 2012.

### Eligibility criteria

Inclusion criteria for review included: documented patient age within 0–5 years; ‘severe anemia’ listed as one of final diagnoses. Exclusion criteria included: patients with a diagnosis of ‘severe anemia’ due to sickle cell disease or surgical conditions or known chronic conditions such as cancer.

### Adherence to guidelines

The evaluation of adherence to guidelines was based on the principles used in the development of Pediatric Admissions Quality of Care (PAQC) score [20, 21]. The PAQC score was specifically designed to assess quality of admission care for malaria, pneumonia and diarrhea in low income countries [20]. As in the PAQC score, we grouped guideline recommended key items that should be performed into specific domains. Items were scored 0 or 1 depending on whether or not they had been carried out as recommended (Table 1). A patient was deemed to have been managed according to guidelines if they scored 1 in each of the pre-determined key items in the domains (Table 1).

**Table 1:** Summary of clinical practice guidelines recommended steps in the management of children with severe anemia and the critical items used to determine adherence to guidelines.

Domain	Itemized CPG^#^ recommended best practices steps	Required critical key items used in this study*
Assessment	Documented severe palmar pallor	
	Triage: Assessed by clinician within 30–60 minutes of arrival to hospital	
	Weight of child measured and recorded	
Classification	Hemoglobin (Hb) level measured.	
	Diagnosis of SA confirmed if Hb ≤ 5.0 g/dl	Pre-transfusion Hb ≤ 5 g/dl
Treatment	Blood transfusion given *promptly* within 2–4 hours of request if indicated.	Received a blood transfusion within 4 hours of request
	Blood given appropriately if indicated *Dose*: *Packed cells 10 ml/kg or whole blood 20ml/kg given over 2–4 hours*	
Follow up (post-transfusion care)	Cause of SA investigated–(malaria tests, complete blood count, iron studies etc)	Relevant laboratory investigations done
	Specific treatment for cause of SA (antimalarials, antibiotics, hematinics, etc)	Received treatment for the cause of SA
	Pre-transfusion Hb done *(repeat transfusion if Hb < 5 g/dl)*.	
	Hematinics given post-transfusion	

Note

^#^ CPG = Clinical Practice Guidelines

* a patient was required to have achieved in all the key critical domains to be considered to have been managed according to clinical guidelines.

### Domains

We considered four critical domains in our review based on the CPG and previously published work [22]: assessment, classification, treatment and follow up. According to CPG, certain specific steps should be performed in each domain in the management of SA children.

#### Assessment

Children suspected to have SA are identified by the clinical sign of severe palmar pallor and are classified at triage as priority or emergency (if they have signs of heart failure like respiratory distress or weak pulse or tender hepatomegaly). Hence suspected SA children should be assessed by a clinician within 30–60 minutes of arrival to hospital. SA children should also have their weights measured and recorded to enable calculation of appropriate volume of blood to be transfused. However, the steps in assessment were not considered critical items for appropriateness of care because palmar pallor is not a specific sign for SA and we could not assess timelines at triage since patient files did not have time of arrival to hospital recorded. Similarly, weight measurement was not included amongst the critical items because the amount of blood transfused were not routinely recorded in patient files.

#### Classification

Children with suspected SA should have level of Hb measured and Hb of ≤ 5 g/dl confirms the diagnosis of SA. Thus a documented pre-transfusion Hb of ≤ 5 g/dl was considered a critical item in the appropriate care of SA children, children with Hb> 5 g/dl or Hb not done were scored as ‘0’.

#### Treatment

A blood transfusion is indicated for Hb ≤ 4 g/dl or if Hb is 4–5 g/dl with signs of heart failure. If indicated, the appropriate grouped and cross-matched blood should be given promptly within 2–4 hours at a dose of 10ml/kg (packed cells) or 20 ml/kg whole blood. Timeliness in receiving blood transfusion is critical in the management of SA and delays in receiving blood transfusions is associated with poor inpatient outcomes [23]. Thus time of initiation of blood transfusion was one of the critical items in the appropriate care of SA. The time of blood request and supply is usually indicated on the request forms and as per CPG patients should receive the blood within 4 hours of request. However, as it was difficult to ascertain precise indications for transfusions as well as the dose and duration of the transfusion, we considered any transfusion for Hb ≤ 5 g/dl as appropriate.

#### Follow up or post transfusion care

Children with SA should have cause of the SA investigated and treated. The most common causes of SA are infections (malaria, bacterial, HIV), micronutrient deficiencies, and helminthic/parasitic infestations [3]. Often these causes co-exist in a child, so ideally several tests and assessments should be done. We expected that every child should have had at least one laboratory test done to investigate the cause of SA and started on specific treatment (anti-malarials, antibiotics, iron supplements). Thus having any relevant laboratory test done and specific treatment given were considered as critical items in the appropriate care of SA children.

Other aspects of post transfusion care such as post-transfusion Hb measurement to assess whether the severe anemia was adequately corrected, and use of hematinics are important but were not considered critical items in the appropriate care. This is because these items are more important for post-discharge rather than inpatient survival.

### Study procedures

Every day from the beginning of June 2016 to the end of March 2017, inpatient files from the pediatric wards of each of the two hospitals with ‘severe anemia’ listed as one of the final diagnoses were identified. The files were collected at the end of hospitalization (discharge or death) and a research assistant used a data extraction form to extract de-identified data on the care the patients received during the hospitalization. The assistants were diploma level nurses who were trained on the use of study tools until they demonstrated competency in data extraction from the files. De-identified data was extracted on basic demographic information, clinical assessments carried out, listed final diagnoses, results of tests (such as Hb, CBCs, malaria tests) ordered for the management of severe anemia, timeliness of treatments given (like blood transfusions, antibiotics, antimalarials), and the outcome of hospitalization (death, discharge or referred). Inpatient death amongst the patients managed as cases of SA was the primary outcome. The death summary book and nurses’ ward reports were checked to verify each reported death and ensure that no SA-related deaths were missed. After the first 4 months (June to September 2016), a quality improvement training intervention was conducted as described below.

### Quality improvement (QI) intervention

A multifaceted QI intervention was implemented at both sites during the study. This included an audit meeting, brief refresher training and smaller unit level meetings to address quality of care issues.

#### Clinical audit meeting

The meeting was attended by nurses, doctors including the pediatricians, medical officers and junior interns working on the children’s ward, laboratory technicians from the hospital laboratory, clinical officers from the outpatient departments, and the administrative head of the hospital (Lira) and the children’s ward (Jinja). A presentation of the results of the level of care provided by the hospital to SA children in the previous four months was presented and discussed. Action points on how to improve care for children with SA were suggested. For each action point, someone was chosen to ensure that the action was implemented.

#### Refresher training

A one-day refresher training was conducted to remind the interns, nurses and doctors working on the paediatric wards on how to triage, resuscitate severely ill patients and how to treat someone with SA. The training consisted of drills and case scenarios using manikins as is done during the ETAT+ provider trainings, with a focus on how to manage children with SA.

#### Smaller administrative meetings

The research team also facilitated discussions between the various departments of the hospital (including the pharmacy, laboratory, blood bank and nursing department). The purpose of the meetings was to update all the staff working in these departments on the gaps in clinical care identified, to discuss ways of addressing these gaps, and to implement the action points from the hospital meeting.

### Sample size

We aimed to review at least 1000 SA files across both sites over the study period with the assumption that 30% (300) of them would be managed according to the guidelines. This would detect a difference in mortality of 50% between those managed versus not managed according clinical guidelines with a level of significance of 0.05 and 90% power. We used a case fatality rate of 14% for SA children not managed according to clinical guidelines [2].

### Statistical analysis

The de-identified data extracted from the patient files were entered in File maker database and analysis was done using STATA version 14.1 statistical software (StataCorp, USA). Clinical characteristics for children managed and those not managed according to clinical guidelines were summarized as proportions and percentages. Performance of the three selected domains were dichotomized as achieved or not achieved. The level of adherence was a binary composite score where a patient was considered to have been managed according to the guidelines if they scored “1” on all key critical items in the domains. The relationship between inpatient outcome (deaths) and various clinical practices was determined using logistic regression. Regression models were also run to test for differences in inpatient outcome amongst study site and period of QI intervention. Both the study site and period of QI intervention were treated as fixed effects. Univariate associations of p value < 0.05 were included in the multivariable model. A backward stepwise approach was used to arrive at the final multivariable model.

### Ethical considerations

Ethical approval including waiver of informed consent to retrospectively review routine inpatient records was granted by the Makerere University School of Medicine Research and Ethics Committee and the Uganda National Council of Science and Technology. Permission to access hospital records for the purposes of this study was granted by the administrators of Jinja and Lira RRHs.

## Results

A total of 1,131 cases were managed for SA in the two hospitals during the study period. The mean age was 2.1 (SD 1.3) years with a slight male preponderance of 654 (57.8%). The mean age, gender distribution, co-morbid diagnoses, blood transfusion rates and duration of hospitalization were similar between children managed and those not managed according to clinical guidelines (Table 2). Amongst children whose time of admission was available, a higher proportion of those managed according to clinical guidelines presented during the day shift, 123/223 (55.2%) vs 176/401(43.9%), while a higher proportion of children not managed according to clinical guideline presented during the night shift, 67/401 (16.7%) vs 22/223(9.9%) (Table 2).

**Table 2:** Demographic, clinical care characteristics, and outcome of children diagnosed as cases of ‘severe anemia’ according whether or not they were managed as per clinical guidelines.

	Managed per guidelines N = 376	Not managed per guidelines N = 755	Total N = 1,131
Age, Mean (SD^†^) in years	2.3 (SD 1.3)	2.1 (SD 1.3)	2.1 (SD 1.3)
Sex, N (% Male)	213 (56.7%)	441 (58.4%)	654 (57.8%)
Referred, N (%)	151 (40.2%)	309 (40.9%)	460 (40.7%)
**Time of admission**	(n = 223)	(n = 401)	(n = 624)
*Day (8*.*01 am– 4*.*00 pm)*	123 (55.2%)	176 (43.9%)	299 (47.9%)
*Evening (4*.*01 pm– 9*.*00 pm)*	78 (34.9%)	158 (39.4%)	236 (37.8%)
*Night (9*.*01 pm to 8*.*00 am)*	22 (9.9%)	67 (16.7%)	89 (14.3%)
First seen at admission by a medical officer	242 (64.4%)	446 (59.1%)	688 (60.8%)
**Co-morbid Diagnosis**			
Severe Malaria (SM)^#^	323 (85.9%)	631 (83.6%)	954 (84.4%)
Septicemia*	166 (44.2%)	310 (41.1%)	476 (42.1%)
Hemoglobinuria*	63 (16.8%)	87 (11.5%)	150 (13.3%)
Severe Acute Malnutrition*	19 (5.1%)	49 (6.5%)	68 (6.0%)
Transfused	376 (100.0%)	749 (99.2%)	1,125 (99.5%)
Duration of hospitalization, Mean (in days)	4.5 (SD 3.2)	4.5 (SD 3.9)	4.5 (SD 3.7)
Inpatient mortality	10 (2.7%)	67 (8.9%)	77 (6.8%)

Note

^†^SD = Standard Deviation

* Clinical diagnoses as assigned on the charts without necessarily being confirmed by laboratory tests

# Malaria diagnosis confirmed by positive blood smear or Rapid diagnostic test.

### Quality of inpatient care

**Fig 1:**
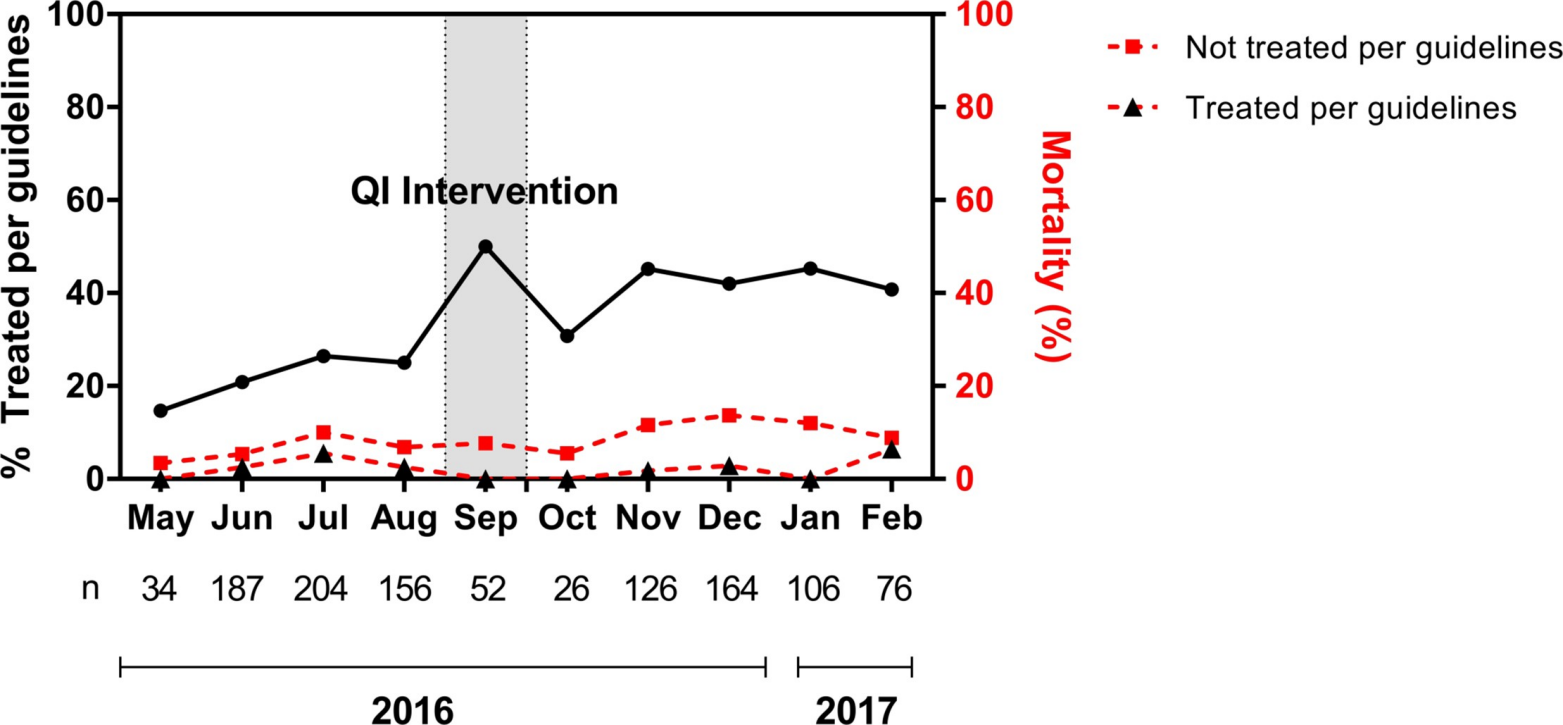
The level of adherence to guidelines over the study period and inpatient mortality rates amongst children managed verses children not managed per guidelines.

Overall 376 (33.2%) of the SA children were managed as per clinical guidelines. Adherence to guidelines was better in Jinja 288 (40%) than Lira 88(21.1%). The difference in adherence was mainly due to higher proportions of Hb measurements and investigations for the cause of SA in Jinja compared to Lira Hospital (Table 3). In each of the hospitals adherence to clinical guidelines was better in the post QI intervention compared to the pre QI intervention period, Lira: 31.1% vs 14.3; Jinja: 48.3% vs 32.9%, respectively (Table 3). Overall a higher level of adherence to clinical guidelines persisted throughout the 4 months of the post QI period (Fig 1).

**Table 3:** The inpatient of care for children 0–5 years managed as cases of severe anemia in Lira and Jinja Hospitals pre and post Quality Improvement (QI) intervention.

Critical Domains of care	Inpatient clinical care indicator	Lira hospital		Jinja hospital	
		Pre- QI^±^ intervention N = 251	Post QI^±^ intervention N = 166	Pre-QI^±^ intervention N = 370	Post QI^±^ intervention N = 344
1. Assessment	Hb done	72 (28.7%)	70 (42.2%)^^^	225(60.8%)	256 (74.2%)^^^
	Hb < 5 g/dl ^a^	56 (22.3%)	59 (35.5%)^^^	172 (46.5%)	181 (52.6%)^^^
2. Treatment	Transfused	250 (99.6%)	164 (98.8%)	368 (99.5%)	343 (99.7%)
	Received blood within 4 hours of request ^b^*	202 (80.5%)	157 (94.6%)^^^	281 (75.9%)	323 (93.9%)^^^
3. Follow up (post-transfusion care)	^#^Cause of SA investigated ^c^	132 (52.6%)	122 (73.5%)^^^	344 (92.9%)	331 (96.2%)
	Malaria test^†^	125 (49.8%)	94 (56.3%)	312 (84.3%)	316 (91.9%)
	CBC^±±^ done	47(18.7%)	69 (41.6%)	169 (45.7%)	176 (51.2%)
	Other tests^††^	5 (1.9%)	33 (19.9%)	38 (10.3%)	19 (5.5%)
	^##^Specific treatment given ^d^	249 (99.2%)	166 (100%)	365 (98.7%)	341 (99.1%)
	Antimalarial	237 (94.4%)	135 (81.3%)	328 (88.7%)	283 (82.3%)
	Antibiotics	182 (72.5%)	155 (93.4%)	279 (75.4%)	270 (78.5%)
	Post transfusion Hb done	20 (7.9%)	41 (24.7%)^^^	238 (64.3%)	237 (68.9%)
	Received hematinics post transfusion	176 (70.1%)	95 (57.2%)	150 (40.5%)	95 (27.2%)
**Overall**	Managed per clinical guidelines**	36 (14.3%)	52 (31.3%)^^^	122 (32.9%)	166 (48.3%)^^^

Note

^a,b,c,d^ Required key clinical indicators in the appropriate management of children with SA

^±^QI = Quality Improvement

^#^ At least one of the investigations done. The cause of SA is multi-factorial so possible to have more than one test.

^##^ Either an antimalarial or antibiotic given. Also possible for a patient to receive both an antimalarial and antibiotic

^†^ Malaria test = Rapid Diagnostic Test or blood smear

^±±^ CBC = complete blood count

^††^ Included urinalysis, stool analysis and HIV serology

* Time to transfusion data available for 1,037 (92.2%) of the patients transfused

** managed per protocol if patient achieved in all selected clinical indicators marked ^a, b, c, d^

^^^ Statistically significant difference between Post and Pre QI intervention

### SA related inpatient mortality

**Fig 2:**
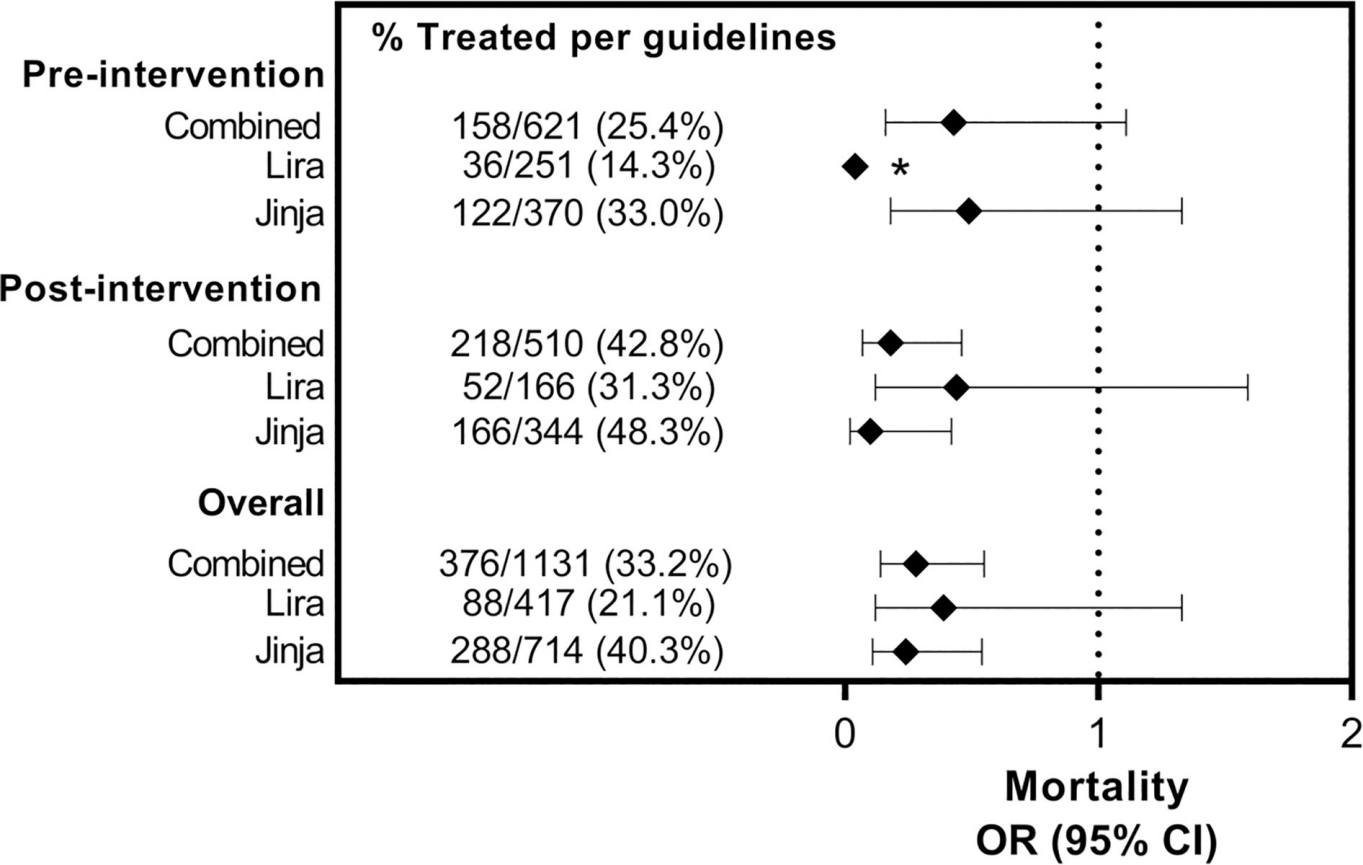
Inpatient mortality rates in children managed verses those not managed according to clinical guidelines in the specified subgroups. * Whiskers crosses null value 1 but is too long to be presented on the graph.

SA children managed according to clinical guidelines had significantly lower inpatient mortality rate compared to those not managed according to clinical guidelines [10/376 (2.7%) vs. 67/755 (8.9%), (*p* < 0.001]. This lower rate of in inpatient deaths in SA children managed according to clinical guidelines was present throughout the duration of the study (Fig 1). The difference in the inpatient mortality between SA children managed versus those not managed according to clinical guidelines tended to widen in the post QI intervention period. SA children managed according to clinical guidelines had reduced risk of inpatient death compared to those not managed according to clinical guidelines, [OR 0.28, (95%, CI 0.14, 0.55), p = 0.001]. The trend of reduced risk mortality in SA children managed as per clinical guidelines was consistent when the data was sub-categorized according to period of QI intervention, and study site (Fig 2).

### Clinical care factors associated with inpatient mortality

The final multivariable logistic regression model showed that clinical care factors associated with reduced risk of death were having pre-transfusion hemoglobin done to confirm diagnosis [OR 0.5 (95% CI 0.29, 0.87)], a co-morbid diagnosis of severe malaria [OR 0.4 (95% CI 0.25, 0.76)], and being reviewed after admission by a clinician [OR 0.3 (95% CI 0.18, 0.59)]. A co-morbid diagnosis of severe acute malnutrition was associated with increased risk of inpatient death [OR 4.2 (95% CI 2.15, 8.22)] (Table 4).

**Table 4:** Clinical management factors from binary and multiple logistic regression associated with inpatient deaths in children managed as severe anemia in Lira and Jinja Hospitals.

	Died	Unadjusted		Adjusted	
Total Study N = 1,131	Yes, n = 77 No, n = 1054	OR (95% CI)	P value	OR (95% CI)	P value
Age less than 2.0 years	49 (63.6%) 482 (45.7%)	2.1 (1.29, 3.36)	0.003	0.4 (0.81, 2.28)	0.249
Pre-transfusion hemoglobin level tested	28 (36.4%) 595 (56.5%)	0.4 (0.27, 0.71)	0.001	0.5 (0.29, 0.87)	0.014
Co-morbid diagnosis of severe malaria	50 (64.9%) 904 (85.8%)	0.3 (0.19, 0.51)	< 0.001	0.4 (0.25, 0.76)	0.004
Co-morbid diagnosis of severe acute malnutrition	18 (23.4%) 50 (4.7%)	6.1 (3.36, 11.2)	< 0.001	4.2 (2.15, 8.22)	< 0.001
Reviewed after admission by a clinician	57 (74.0%) 941 (89.3%)	0.3 (0.19, 0.59)	< 0.001	0.3 (0.18, 0.59)	<0.001
Co-morbid diagnosis of Hemoglobinuria	3 (3.9%) 147 (13.9%)	0.3 (0.08, 0.80)	0.020	0.4 (0.13, 1.42)	0.166
Cause of SA investigated ^a^	53 (68.3%) 876 (83.1%)	0.4 (0.27, 0.75)	0.002	0.6 (0.34, 1.12)	0.114
Unspecified febrile illness	41 (53.3%) 435 (41.3%)	1.6 (1.02, 2.56)	0.041	1.5 (0.92, 2.54)	0.099
Received blood within 4 hours of request	63 (81.8%) 900 (85.4%)	0.8 (0.42, 1.41)	0.396		
Given specific treatment ^b^	75 (97.4%) 1046 (99.2%)	0.3 (0.06, 1.37)	0.118		
Admitted during day time (8.00 am to 4.00 pm)	21 (56.8%) 278 (47.4%)	1.5 (0.75, 2.85)	0.269		
Site (Jinja or Lira Hospital)	47 (61.0%) 667 (63.3%)	0.9 (0.57, 1.46)	0.694		
Intervention (Pre or post QI intervention)	39 (50.7%) 471 (44.7%)	1.3 (0.78, 2.02)	0.311		

Note

^a^ Investigations included malaria tests (blood smear or RDT), complete blood count, urinalysis, stool analysis and HIV serology)

^b^ Specific treatment included antimalarials and or antibiotics

## Discussion

We evaluated the effect of adherence to clinical guidelines on inpatient mortality among children managed for SA. Our study found that adherence to clinical guidelines reduced inpatient mortality in children with suspected SA by 72%. Similarly, for children with severe acute malnutrition, a recent meta-analysis also found that management according to WHO guidelines reduced inpatient deaths by 41% [24]. Taken together these findings highlight the important role that adherence to clinical guidelines plays in determining inpatient outcomes for critically ill children in resource-limited settings.

In this study, we were able to implement a QI training intervention resulting in a variation in the level of adherence to clinical guidelines during the study period. Overall and at whatever level of adherence, SA children managed according to clinical guidelines consistently had lower inpatient deaths than those not managed according to clinical guidelines. In follow-up studies in similar settings where guidelines were strictly adhered to, severe malarial anemia related inpatient mortality was reported to have been as low as 0.5% [25]. Therefore ensuring that children with suspected SA are managed according to existing clinical guidelines may be key in reducing the high SA-related inpatient mortality in resource limited settings.

In contrast, we note the high mortality in the children not managed according to guidelines especially in the post intervention phase. Although the reason for this is not clear we found that a higher proportion of the children not managed according to clinical guidelines presented during the night shift when staffing levels are usually low and the laboratories are not fully functional. It is therefore possible that these children were inadequately assessed and started on inappropriate treatment and hence the poor outcomes. There is therefore need for health workers to adhere to clinical guidelines in the care for common clinical conditions like SA. Simple QI interventions like in-service training and clinical audits can be effective in improving adherence to clinical guidelines [17, 18, 26]. In our study the QI intervention led to better flow of patients, more timely intervention and better utilization of the laboratory services.

However, despite implementation of a QI intervention, the overall level of care remained low with only 40% of children being managed according to clinical guidelines. This highlights the complexity of enacting substantial and long-lasting change with QI interventions in health systems that face several barriers to providing effective care. Irimu et al. [22] evaluated the effects of the implementation of QI initiatives on the quality of care for three diseases at a tertiary unit in Kenya over a three-year period. Using QI initiatives that included training of health workers on evidence based clinical guidelines, audit meetings and strengthening support supervision by use of champions, the quality of care improved from a pre-intervention level of 13% to a post-intervention level of 33% of indicators achieving performance over the acceptable 70%. The tasks that required team work (e.g. administration of penicillin, assessment and classification of severe malnutrition) did not show improvement over the study period. These results suggest that QI interventions that focus on training of health workers without addressing other critical determinants related to the provision of care may be insufficient [27]. There is therefore a need for consideration of broader implementation strategies that target institutional and organizational aspects of service [27].

For the management of SA children, interventions need to focus on improving pre-transfusion hemoglobin testing, close monitoring of patients during admissions and proper management of common co-morbid conditions like severe malaria and severe acute malnutrition. SA children with documented pre-transfusion Hb levels and those who were reviewed by the clinician after admission had reduced risk of inpatient deaths. Perhaps clinicians were more aggressive if they knew the Hb, advocating for more timely receipt of blood products for transfusion and other essential treatment measures. In contrast for the children without pre-transfusion Hb, valuable time may have been lost in trying to manage the severe anemia instead of establishing the correct diagnosis and initiating appropriate treatment. Similarly, reviewing patients after admission is good clinical practice as it enables clinicians to address any complications that might have been missed during the admission or arisen since hospitalization.

The other important factors that we found to be associated with inpatient mortality were the co-morbid diagnoses associated with SA. Children with a diagnosis of severe malaria had a better inpatient outcome than those with non-malarial diagnosis (5.2% vs 15.3%). Amongst children diagnosed with malaria, those with a laboratory confirmed diagnosis had lower inpatient mortality than those treated for malaria without laboratory confirmation (4.2% vs.9.8%). On the other hand a co-morbid diagnosis of severe acute malnutrition, as has been previously reported [2, 28], was associated in this study with a six fold increase in the odds of inpatient death. The different co-morbid diagnoses in SA are associated with different in patient outcomes. These further highlight the need for clinicians to carry out tests to establish the etiological factors associated with SA in order to provide appropriate treatment. Further studies are needed to determine the role of the etiological factors associated with anemia on inpatient mortality.

The study had some limitations. The determination of the adherence to clinical guidelines was not based on a validated tool. The evaluation of adherence to clinical guidelines is a complex and multifaceted process due to the several guideline-recommended steps in the management of each disease condition. As such there are no simple standardized tools available. In this study we assessed adherence to guidelines based on the principles used in the (PAQC) [20]. The critical domains assessed and the scoring rubric adopted for this study are therefore similar to those used in other studies [12, 21, 22].

The other limitation was that given that this was a retrospective chart review of routine data that is usually of poor quality with missing data and or undocumented procedures and tests. So our study might have underestimated the level of care that the SA patients received. It is also possible that the reported improvement in adherence after the QI training intervention was simply due to better documentation of clinical intervention and laboratory tests done in the post intervention. As for missing data, we selected as critical key items variables that were fairly well documented in the assessment of level of adherence to guidelines. Variables that had lots of missing data like timeliness of triage, appropriate doses of blood and medicines were not considered. Inpatient deaths which was the main outcome is a major event that was also well documented.

We also note that the co-morbid diagnoses associated with SA were not fully investigated and their proper management not assessed in this study. It is therefore possible that the difference in mortality rates seen in this study was due to the difference in management of the co-morbid diagnoses associated with the SA and not necessarily due to the level of SA related inpatient care received. Regardless, the trend of a consistently higher death rate in children not managed according to clinical guidelines in both sites, before and after QI interventions support the importance of the role of proper management of childhood conditions on inpatient survival.

In conclusion, we found that adherence to clinical guidelines in the management of children with severe anemia is associated with reduced inpatient mortality in the two referral hospitals in Uganda. Efforts to reduce severe anemia related inpatient mortality in resource-limited settings should focus on training and supporting health workers to adhere to clinical guidelines. Future studies should evaluate the role of co-morbid diagnoses on SA related inpatient mortality.

## Supporting information

S1 File Dataset.dta: Minimum dataset for the data presented in the manuscript. (DTA)

S2 File Codebook.xlsx: Codebook for the dataset. (XLSX)
